# Photographic measurement of upper-body sitting posture of high school students: A reliability and validity study

**DOI:** 10.1186/1471-2474-9-113

**Published:** 2008-08-20

**Authors:** Sjan-Mari van Niekerk, Quinette Louw, Christopher Vaughan, Karen Grimmer-Somers, Kristiaan Schreve

**Affiliations:** 1 Stellenbosch University, Cape Town, The Republic of South Africa; 2 University of Cape Town, Cape Town, The Republic of South Africa; 3 University of South Australia, South Australia, Australia; 4 Division of Physiotherapy, Department of Interdisciplinary Health Sciences, Faculty of Health Sciences, Stellenbosch University, P O Box 19063, Tygerberg, 7505, The Republic of South Africa

## Abstract

**Background:**

All the reported measures of sitting posture, as well as photographs, have one flaw, as these measures are external to the body. These measures use calculations from external bony landmarks to estimate spinal posture, on the understanding that what is being measured externally reflects the shape, health and performance of structures of the underlying spine. Without a comparative measure of the relative position of the structures of the spine, the validity of any external spinal posture measure cannot be established. This paper reports on a study which tests the validity of photographs to measure adolescent sitting posture.

**Methods:**

The study was conducted in a laboratory at the Department of Human Biology, University of Cape Town. A random sample of 40 adolescents were recruited from the Cape metropolitan schools, to detect differences of three degrees or more between the repeated measures of upright, normal or slouched posture (photographs) and between the posture photographs and LODOX measures. Eligible participants were healthy male and female subjects aged 15 or 16 years old, in Grade 10, and who were undertaking Computer or Computype studies at their schools. Two posture measurement tools were used in the study, namely: Photographs were taken using the Photographic Posture Analysis Method (PPAM) and Radiograph*s *were taken using the LODOX (LODOX (Pty) Ltd) system. Subjects' posture was assessed in simulated computer workstations. The following angles were measured: the sagittal head angle, cervical angle, protraction/retraction angle, arm angle and the thoracic angle.

**Results:**

Data from 39 subjects (19 males, 20 females) was used for analysis (17 15-year-olds (7 boys and 10 girls), 22 16-year-olds (12 boys and 10 girls)). All but one photographic angle showed moderate to good correlation with the LODOX angles (Pearson r values 0.67–0.95) with the exception being the shoulder protraction/retraction angle Pearson r values. Bland Altman limits of agreement illustrated a slight bias for all angles. The reliability study findings from repeated photographs demonstrated moderate to good correlation of all angles (ICC values 0.78–0.99).

**Conclusion:**

The findings of this study suggest that photographs provide valid and reliable indicators of the position of the underlying spine in sitting. Clinically it is important to know whether a patient is showing true progression in relation to a postural intervention. Based on the results of this study, the PPAM can be used in practice as a valid measure of sitting posture.

## Background

The prevalence of back pain among high school students has been regularly reported to be an international public health concern [1]. However, given the high frequency of research into adult back pain, adolescent back pain has a much lower research profile. In the small amount of available research, a high prevalence of back pain has been reported in the early teenage years, which then increases each year until the late teens [2-4]. In developed countries, the lifetime prevalence of back pain in 15-year-olds exceeds 50% [3]. Whether there is a similar trend among high school students in developing countries such as South Africa is yet to be established.

There is limited but consistent research which indicates that many adolescents reporting frequent back pain become adults reporting back pain, perhaps because causal mechanisms and pain sensitisation become established during the formative years [1,5]. Given the high costs to the individual and to society of adult back pain [6], minimising its prevalence by understanding causal mechanisms of precursor adolescent back pain would seem to be a public health priority.

A number of causal mechanisms have been proposed for adolescent back pain, including carriage of heavy school bags, rapid bony growth, inadequate fit of furniture to body size, poor muscle strength, poor motor control, balance and coordination, and poor posture [5,7-9]. However, despite the interest in adolescent back pain, its causes are far from well understood. Sustained and poor sitting postures have been identified as important risk factors for back pain in adults [10,11]. A common reason for adults to sit for long periods of time in poor postures is when using computers [12]. Healthy computer use involves good workstation design features such as appropriate fit of body size to chair and desk height, screen angle and height, and keyboard arrangement, as well as the amount of time spent at the computer. Extended computer use has thus been proposed as a reason for adult back pain [13,14]. Computer use is increasingly common among high school students around the world, although whether it constitutes a risk for adolescent back pain has not been established [15,16]. Prior to testing any association between computer use and back pain, a reliable and valid measure of sitting posture is required. Any valid posture measurement tool must be able to detect postural abnormalities that could place abnormal stress on spinal structures.

We undertook a systematic review of published research, which reported on sitting posture measurement tools. We identified nine relevant papers describing only three measurement approaches (goniometer [17-19], inclinometer [20-23] and flexicurve [24,25]). None of these approaches has been validated for adolescents (high school students). We found no papers in this systematic review on use of photographs to measure sitting posture, although photographs have been reported as a measure of adult, adolescent and children's standing posture [26-28]. Given the reported reliability and efficiency of photographs, the longevity of digital records, and the cost-effectiveness of digital photographs to measure standing posture in adults and children, it is feasible that they would also be appropriate to measure sitting posture in adolescents.

All the reported measures of sitting posture, as well as photographs, have one flaw. These measures are external to the body, that is, they use calculations from external bony landmarks to estimate spinal posture, on the understanding that what is being measured externally reflects the shape, health and performance of structures of the underlying spine. Without a comparative measure of the relative position of the structures of the spine, the validity of any external spinal posture in humans is often difficult to establish and may not give an accurate interpretation of true spinal alignment. The only trustworthy measure of the position of spinal structures is Radiography [24,29]. To date, however, little research has been undertaken to validate external posture measurement methods with Radiographs into healthy spinal posture, and this may largely be because of the ethical and health implications of subjecting healthy spines to irradiation [30]. These concerns are, if possible, even more important for adolescents, given the potential influence of irradiation on growing systems and organs [30].

Recently a low dose Radiograph was developed in South Africa. The LODOX (LODOX (Pty) (Ltd) (a digital radiography device) was developed by De Beers as a safe Radiograph security scanner for the detection of smuggled diamonds. LODOX conducts a full body scan in 13 seconds, with smaller areas requiring proportionately less time [31]. On average, the mean conventional dose of radiography is 0.573 R (5.73 mGy) while the mean digital dose (LODOX) is 0.033 R (0.33 mGy), 5.9% of the dose of the conventional Radiograph [31]. Low dose radiograph systems provide population-applicable, 'Gold Standard' radiographic approach for measuring spinal segmental posture in healthy individuals.

This paper reports on a study which tests the validity of photographs to measure adolescent sitting posture. The aim of the project is to correlate the postural angles of the photographs with LODOX images, for three types of adolescent sitting spinal postures (slouched, upright or normal).

## Methods

### Ethics

Ethical approval was obtained from the Committee for Human Resources at Stellenbosch University and the Western Cape Department of Education. Written informed consent was obtained from all students, and their parents or legal guardians.

### Setting

The study was conducted in a laboratory at the Department of Human Biology, University of Cape Town.

### Sample size

Sample size calculations were based on previously reported variability in sitting posture angles in healthy adults [32,33]. As little normative data was available on healthy adolescent sitting posture, this sample calculation was an estimate only. A sample of 40 was proposed (power 80%, alpha 5%) to detect differences of three degrees or more between the repeated measures of upright, normal or slouched posture (photographs) for the reliability study and between the posture photographs and LODOX measures for the validity study.

### Sample

The population, from which the study sample was selected, comprised high school students from the Cape Metropolitan Region, Cape Town, South Africa. The Cape Metropolitan Region is divided into four educational management regions. One school from each region was selected by a statistician independent of the study, using a random numbers table. Eligible participants were healthy male and female subjects aged 15 or 16 years old, in Grade 10, and who were undertaking Computer or Computype studies. Eligible subjects in the selected schools were asked to volunteer to participate in the study. Subjects were excluded if they experienced any recent musculoskeletal pain or illness, which could compromise their ability, to assume upright, slouched or normal sitting posture on the day of data collection. These subjects were identified using a pain questionnaire that has been extensively validated for this population of high school students using computers. This questionnaire was administered prior to the commencement of validity and reliability testing [34].

### Measurement tools

Two posture measurement tools were used in the study.

*1. Photographs *were taken using the Photographic Posture Analysis Method (PPAM). This method consisted of a digital camera (Fujifilm Finepix X5100), Intellect software (Version 1.1.4), reflective markers (see later section for details) and a computer for downloading images (Windows 2000 or XP compatible).

**Figure 1:**
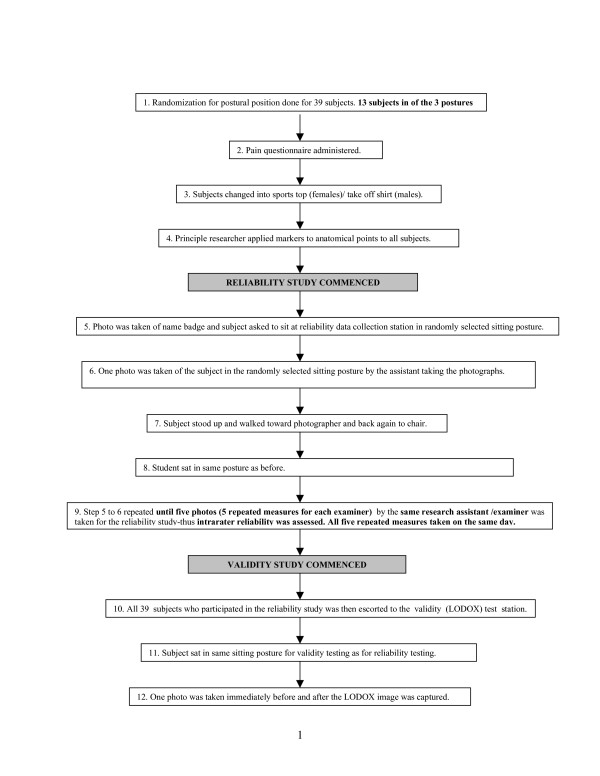
Data collection procedure for each subject (reliability and validity studies).

*2. *Radiograph*s *were taken using the LODOX (LODOX (Pty) Ltd) system (see Figure 1).

### Test purposes and set-up

To test the validity of the photograph compared with the Radiograph, the testing station consisted of the LODOX surrounding the computer workstation, and one digital camera mounted on a tripod outside the LODOX. The LODOX system captured an image of the upper part of the body (T8 to head). The digital camera was positioned to capture the same spinal area as the LODOX. To test the reliability of sitting postures using photographs, the same workstations were set up outside the LODOX.

### Posture measurement set-up

Subjects' posture was assessed in simulated computer workstations. The chair height and seat pan depth were selected based on the findings of an evaluation of school workstations in order for the chairs to simulate the typical chairs used in the schools [35]. Subjects could not adjust the chair position to suit their personal preference, as the current chairs in the schools are not adjustable. The chair height was between 440 mm and the seat pan depth was between 380 mm.

### Data collection procedures for the reliability and validitystudies

The same 39 subjects participated in both the reliability and validity studies. The subjects and both studies were conducted on the same day for specific subject. Figure 1 outlines the data collection procedure for both the reliability and validity studies.

### Subject preparation and positioning

Anatomical markers were placed on all subjects by the one researcher, to identify seven external landmarks in photographs. These landmarks were randomly checked by another researcher (QL) to confirm their accuracy of placement. Prior to placement, the relevant areas of the subjects' skin were wiped with alcohol to facilitate good contact between the reflective markers and the skin. Golem retro-reflective markers were applied to the lateral canthus of the eye, the tragus of the ear, the spinous process of C7 [35], the midpoint of the superior border of the manubrium, T8 and the lateral epicondyle of the elbow [15]. Both C7 and T8 markers were placed on extension sticks to allow for better visibility by the camera. All markers were placed on the subjects' dominant side and were not removed until testing was completed. Photographs and radiographs were taken from the dominant side. The markers were checked between each photograph and radiograph measure to ensure that they were in place, and accurate.

### Posture estimation

**Figure 2:**
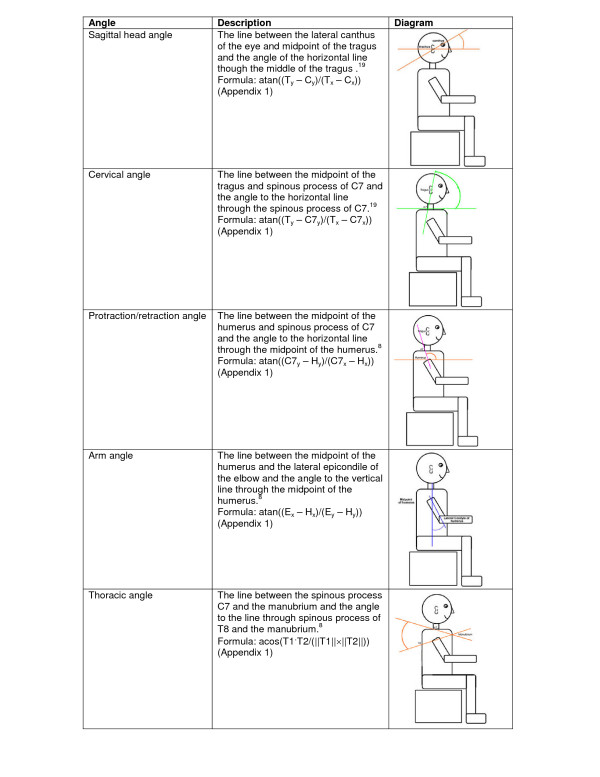
Diagrammatic representation of the angles measured.

Five postural angles were calculated from the LODOX images and the photographs (outlined below and illustrated in Figure 2).

a) The sagittal head angle indicates the position of the head relative to the neck [28]. This angle is commonly affected by computer usage [17]. McEvoy and Grimmer (2006) reported that a decrease in this angle reflects a "poking-chin" posture [28,36].

b) The cervical angle is the measure of the forward-head position, which is a useful clinical marker of mid/lower cervical spine posture [28].

c) The protraction/retraction shoulder angle was measured using the method by Szeto *et al. *(2002) [16].

d) The arm angle was not measured in previously published studies. However, we have decided to measure the arm angle as it may be associated with the degree of shoulder protraction/retraction angle.

e) The thoracic angle was also measured in the manner described by Szeto *et al. *(2002) [15,16]. Unfortunately few measures for this spinal section are reported in the literature.

### Camera positioning

For all tests, the digital cameras (flash on) were mounted on tripods and placed 2 metres away from the chair on which the subject was positioned. The cameras were positioned so that all anatomical markers were detectable in the one image.

### Test protocols

Approximately 12 subjects were tested per day. When they attended the testing session, subjects were randomly allocated to one of three sitting postures (slouched, straight or 'normal' (normal) sitting), as outlined in Figure 1. Subjects who had to assume the slouched posture were given the following instructions to "lean with your arms on the table with your back bend forwards", subjects who had to assume the straight posture were given the instructions to "sit up straight with head, shoulders and hips in line", while subjects who assumed the normal posture were given the following instructions "sit as you would normally sit in front of a desktop computer" subjects were given two to three practice opportunities to accommodate to the assigned posture. Subjects were instructed to assume the same allocated sitting posture for all tests. The use of three postures served to ascertain whether the PPAM could validly and reliably test postural angles through sitting posture range.

### Data capture from images

The photographic and radiographic data was imported to a laptop via a USB data-transfer cable and Intellect 1.1.4 software (DVT Corporation). The principal researcher digitized all photographic and radiological data in order to calculate the angles. The Intellect 1.1.4 software functions are 'detecting and following a marker', 'circle fitting', 'constructing lines' and 'measuring angles'.

#### Step 1

**Figure 3:**
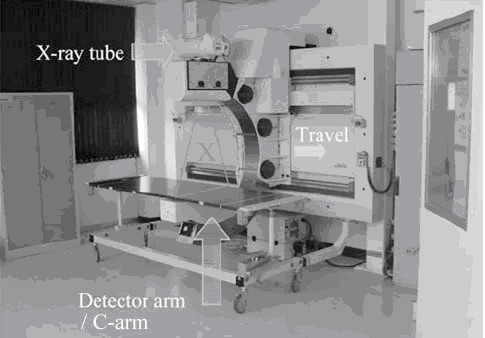
LODOX machine.

To digitize the information of the Lodox images (see figure 3), the operator electronically placed a marker on the respective bony landmarks (spinous processes) of C7 and T8 in order for the software to detect the bony point. The rest of the markers could be used as they were as they were already placed on bony landmarks. Detecting and following a marker was the most complex function during the digitizing process. The software recognized the markers by defining the edges of the image. The user was required to 'teach' the software how to recognize the marker. The shape of the marker is 'learned' by the software by defining the edges in the image. Software learning refers to an automated memory of the software to process the information as done before.

#### Step 2

**Figure 4:**
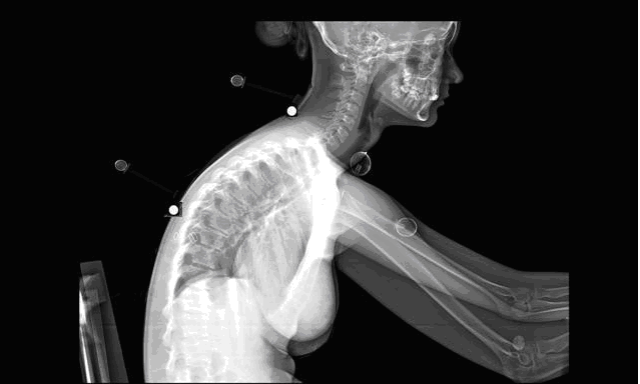
LODOX image.

Once the software detected the marker, the next step in the photographic digitising process was to calculate the centre of the marker. This was done by applying edge detection on the border of the marker and thereafter a circle was fitted through the edge points. Provided that the markers could be detected accurately, the calculation of the angles for a series of images could be automated (See Figure 4)

#### Step 3

##### Calculation of the angles

The system was programmed for the first image of each participant, and additional software (DVT Reader) was developed to apply the digitizing process described above to the full set of photographic images, instead of only a single image, in order to calculate the angles much faster than with the original Intellect 1.1.4 software. A co-author (KS) and another engineer developed the additional software. The angles were calculated using basic trigonometry. The (X, Y) positions of the markers are provided, as well as the length of the stems, where applicable. An example of how an angle was calculated is provided in Appendix 1.

##### Statistical comparisons

Descriptive and comparative statistics were calculated to determine differences and correlations between measurements. The mean and standard deviation for each angle from photographs and radiographs was calculated using Microsoft Excel (2002) and SPSS Viewer Version 14 software, for each of the sitting postures. Concurrent validity was estimated as Pearson r correlation coefficients, calculated between the mean angles from the two photographs, and the LODOX measures for each posture (normal, slumped and upright). Bland Altman, with the 95% limits of agreement equivalent to the mean difference ± 2 SD was also calculated to compare the angle values of the photographs and radiographs. Reliability was calculated between the angles from the five repeated photographs. Five repeated photos were taken for reliability study, this excluded photos taken for the validity study. Reliability was determined from the interclass correlation coefficients (ICCs) by means of the 2-way model and Standard Error Measurement (SEM) [37], with the strength of the ICCs interpreted as <0.50 = poor, 0.50 < 0.75 = moderate, 0.75 < 0.90 = good and > 0.90 = excellent. The ICC and SEM convey different information about reliability of a measure.

The ICC provides information about a measure's capacity to differentiate change within subjects, whereas the SEM quantifies the error in the same units as the original measurement and therefore provides meaningful information about the reliability of the measurement.

## Results

Although 40 subjects consented to participate, one subject refused to undress her right (dominant) side due to burn scars. The data from 39 subjects (19 males and 20 females) were thus used for analysis for the reliability and validity studies. A total of seventeen 15-year-olds (7 boys and 10 girls) and twenty-two 16-year-olds (12 boys and 10 girls) were examined. Table 1 reports participants' age, gender and posture allocations.

**Table 1:** The students' age, gender and posture

	**15-year-olds**		**16-year-olds**		
		
**Posture**	**Male**	**Female**	**Male**	**Female**	**Total**
**Slouched**	2	5	4	2	**13**
**Upright**	3	2	4	4	**13**
**Normal**	2	3	4	4	**13**
**Total**	**7**	**10**	**12**	**10**	**39**

The findings of this study suggest that photographs provide valid and reliable indicators of the position of the underlying spine in sitting.

### Validity

Table 2 reports the Pearson r values comparing the LODOX measures with the mean values from two photographs, of the five PPAM angles in each of the randomly allocated postures. All photographically captured angles (except for the protraction/retraction angle) demonstrated strong correlation with LODOX angles, with Pearson r correlation values of at least 0.84. The protraction/retraction angle in the normal sitting posture demonstrated the lowest Pearson r correlation value overall, even thought this was still a moderate correlation.

**Table 2:** Validity findings (Pearson r values)

**Validity**	**Sagittal head angle**	**Cervical angle**	**Protraction/retraction angle**	**Thoracic angle**	**Arm angle**
All angles measures from validity photos and LODOX	0.84	0.89	0.89	0.92	0.79
**Upright (n = 13)**	0.73	0.89	0.88	0.81	0.76
**Normal(n = 13)**	0.97	0.85	0.48	0.93	0.86
**Slouched (n = 13)**	0.84	0.79	0.90	0.93	0.66
**Female (n = 20)**	0.67	0.90	0.73	0.95	0.75
**Male (n = 19)**	0.92	0.89	0.87	0.86	0.87

### Bland Altman limits of agreement

Bland Altman limits of agreement are demonstrated in Table 3. The Bland Altman method revealed a small bias of -1.12° for the cervical angle, -1.56° for the head angle, -1.98° for the shoulder protraction/retraction angle, -3.76° for the arm angle and -1.12° for the thoracic angle.

**Table 3:** Bland Altman Limits of Agreements

	**Limits of agreement ± 2 SD**
**Sagittal head angle**	-7.04–3.93
**Cervical angle**	-8.04–6.73
**Protraction/retraction angle**	-11.45–15.41
**Thoracic angle**	-8.61–6.37
**Arm angle**	-10.84–3.32

### Reliability results

Table 4 reports the descriptive statistics for each of the angles measured in the three sitting postures. The protraction/retraction angle demonstrated the largest variability (largest SD) in each sitting posture.

**Table 4:** The mean, SD and range values of the angles

**Angles**	**Normal**			**Upright**			**Slouched**		
	
	**Mean**	**SD**	**Range (degrees)**	**Mean**	**SD**	**Range (degrees)**	**Mean**	**SD**	**Range (degrees)**
**Sagittal head angle**	20.05	7.84	0 – 34.70	19.99	8.15	0.90 – 34.40	10.28	10.68	(-)15.90 – 34.20
**Cervical angle**	47.66	9.75	21.90 – 62.90	52.72	11.18	22.30 – 71.30	21.49	27.57	(-)34.10 – 53.40
**Protraction/retraction angle**	130.21	25.77	65.30 – 178.70	124.76	20.36	76.50 – 159.80	145.68	20.62	103.70 – 208.70
**Thoracic angle**	63.25	8.57	49.50 – 89.20	61.37	11.76	40.80 – 97.60	61.46	8.88	39.30 – 78.10
**Arm angle**	23.46	12.75	(-)5.00 – 50.30	24.21	12.09	3.30 – 60.90	32.72	10.34	14.50 – 48.80

All angles calculated from the repeated photographs demonstrated moderate to good agreement (See Table 5). Neither sitting posture nor gender significantly influenced the reliability of the angles calculated from the repeated photographs.

**Table 5:** Reliability Findings: ICC's and SEM values of all angles, postures and genders

**Reliability**	**Sagittal head angle**	**Cervical angle**	**Protraction/retraction angle**	**Thoracic angle**	**Arm angle**
95% Lower and Upper interval	0.82 – 0.96	0.86 – 0.96	0.74 – 0.93	0.60 – 0.97	**0.95–0.94**
All angles (5 reliability photos)	0.98	0.98	0.94	0.96	0.99
**Upright (n = 13)**	0.97 (0.93–0.99)	0.98 (0.55–0.93)	0.92 (0.79–0.93)	0.97 (0.83–0.99)	0.99 (0.97–0.99)
**Normal (n = 13)**	0.97 (0.92–0.97)	0.78 (0.56–0.94)	0.91 (0.78–0.92)	0.92 (0.84–0.98)	0.98 (0.96–0.98)
**Slouched (n = 13)**	0.98 (0.99–0.95)	0.98 (0.96–0.98)	0.99 (0.97–0.99)	0.97 (0.93–0.99)	0.98 (0.95–0.98)
**Female (n = 20)**	0.96 (0.92–0.96)	0.99 (0.98–0.99)	0.94 (0.88–0.97)	0.94 (0.89–0.97)	0.98 (0.97–0.99)
**Male (n = 19)**	0.99 (0.97–0.99)	0.96 (0.91–0.98)	0.95 (0.88–0.96)	0.97 (0.94–0.98)	0.97 (0.95–0.98)
**SEM **(in degrees)	3.50	8.06	11.09	4.04	3.33

## Discussion

This paper reports the first known research to report on the validity of photographs of adolescent sitting posture, based on comparison with 'Gold Standard' Radiograph measures, the LODOX. The LODOX measures in this study provide unique information on the position of the spine in healthy adolescents sitting in a range of positions at computer workstations. The comparison between photographs and LODOX establishes, for the first time, the validity of photographs of external landmarks in measuring posture. Prior to this study, photographs have only been assumed to be representative of underlying spine position. Bland Altman analysis demonstrated a small bias and relatively wider limits for the shoulder protraction/retraction and arm angles. We have proposed that the circle fit function of the software may explain these variations. Given this explanation and the moderate to strong correlations between angles calculated from LODOX and digital photographs for all sitting postures, angles calculated from anatomical landmarks from photographs may be usefully proposed as a measure for gross estimates of spinal curvature. However spinal geometry still cannot be inferred from external postural analysis and should also be addressed in future studies.

### Photographs

Based on the strong correlations between angles calculated from LODOX and digital photographs for all sitting postures, angles calculated from anatomical landmarks from photographs can be proposed as an alternative 'Gold Standard' for estimating sitting posture, on the assurance that they allow gross estimates of spinal curvature. Repeated angles calculated from photographs of subjects in the three sitting postures were also reliable, which suggests that one photograph only, taken in any sitting position, would provide an accurate representation of spinal posture for that individual. The PPAM method is cost and time-efficient, is non-invasive and incurs no exposure to radiation. Thus it is an ideal tool for use in large epidemiological studies of sitting posture in school settings.

### Measurement issues

The researchers experienced difficulty in detecting the sticks on which the external markers for C7 and T8 were placed, with the Intellect 1.1.4 Software. We recommend that these sticks be covered in retro-reflective material in future studies, as this will ensure easier detection of the angle against which the marker is positioned on the body on the Radiograph image.

### Choice of angles

All angles assessed in this study appear to be useful indicators of different aspects of stresses on the cervical and thoracic spine in sitting. The variability in the five angles across the three sitting postures was sufficiently large to enable future research to investigate issues such as the association between reported pain, muscle strength and length, and low, medium and high angles in each anatomical area.

### Angles

The values for the sagittal head angle, cervical angle and protraction/retraction angle were similar to those published by Szeto et al [15,16], which suggested that adolescent angles were similar to adult angles, and that different sitting postures could be captured by the range of angles from photographs. The cervical angle demonstrated moderate reliability in the normal sitting posture with the second highest SEM value of all angles measured. The range for normal sitting posture is very wide compared to the upright and slouched postures which are more repeatable as they represent end of range positions. Thus, students were more likely to resume an extreme postural position (such as slouch or upright), than to accurately repeat the precise position of the spine and body segments in the normal posture range. The arm angle however, has not been reported on in the current published literature. We believed that it was an important angle as it may confound the values of the shoulder protraction/retraction angles. Shoulder protraction/retraction may be biomechanically affected by the position of the arm in glenohumeral flexion and extension [38]. This functional link could occur because of the structural linkage of multiple ligaments and muscles crossing the shoulder girdle complex [38]. The arm angle was thus measured to understand potential confounding effects in shoulder protraction/retraction angle reliability values. Both the arm angle and the protraction/retraction angle showed large variation in range in all three of the measured postures. This might be because subjects were not given a standardised position for their hands on the desks. We propose that for future studies, subjects keep their hands on actual keyboards for the duration of testing, as this might decrease the large variance in the arm angle and the protraction/retraction angle range. The thoracic angle showed very little change in the angle between postures. This may be because the thoracic region is the most inflexible region of the vertebral column. Therefore, since the body usually follows the path of least resistance, it may explain why relatively less movement was noted between the three postures.

### Clinical application

Clinically it is important to know whether a patient is showing true progression in relation to a postural intervention. Based on the results of this study, the PPAM can be used in practice as a valid measure of sitting posture.

## Conclusion

The findings of this study suggest that photographs provide valid and reliable indicators of the position of the underlying spine in sitting. Clinically it is important to know whether a patient is showing true progression in relation to a postural intervention. Based on the results of this study, the PPAM can be used in practice as a valid measure of sitting posture.

## Appendix 1

**Table 6:** Declaration of Symbols

C7'_x_, C7'_y_	X coordinate of C7 marker
T8'_x_, T8'_y_	X coordinate of T8 marker
M'_x_, M'_y_	X coordinate of Manubrium marker
θ_C7_	Smallest angle between horizontal and C7 marker stem
θ_T8_	Smallest angle between horizontal and T8 marker stem
θ_M_	Smallest angle between horizontal and Manubrium marker stem
L_C7_	Length of C7 stem
L_T8_	Length of T8 stem
L_m_	Length of Manubrium stem

The first step is to calculate of the actual position of C7, T8 and the manubrium. The positions are as follows:

C7_x _= C7'_x _+ L_C7_cos(θ_C7_)

C7_y _= C7'_y _- L_C7_sin(θ_C7_)

T8_x _= T8'_x _+ L_T8_cos(θ_T8_)

T8_y _= T8'_y _- L_T8_sin(θ_T8_)

M_x _= M'_x _- L_m_cos(θ_C7_)

M_y _= M'_y _- L_m_sin(θ_C7_)

Now the angles can be calculated. We denote vectors in bold. The dot product is denoted with "·". The vector norm is denoted with "|| ||".

Thoracic Angle

Let **T1 **be the vector from the manubrium to C7:

**T1 **= {C7_x _- M_x_; C7_y _- M_y_}

Let **T2 **be the vector from the manubrium to T8:

**T2 **= {T8_x _- M_x_; T8_y _- M_y_}

Then the thoracic angle is: acos(**T1·T2**/(||**T1**|| × ||**T2**||))

## Study limitations

The height and weight of the students were not measured in this study, but may be useful in future studies which also incorporate chair compatibility. A further limitation was that markers were placed manually on the C7 and T8 spinous processes of the spine and reliability of the manual placement of these markers were not tested. The circle fit process is not always accurate and therefore we recommend further development of the data processing software where this aspect of the data processing is standardised electronically.

## Recommendations for future studies

Photographs measured using the PPAM system are valid indicators of adolescent sitting posture. When given standard instructions regarding assuming a sitting posture, subjects' posture is also reliable, when measured by repeated photographs.

## Competing interests

The authors declare that they have no competing interests.

## Authors' contributions

SVN conducted the study and drafted the manuscript. QL conceptualised the project, assisted with data collection and revised the manuscript. KGS assisted with statistical advice, study design assisted in writing the manuscript. CV assisted with project conceptualisation and LODOX imaging. KS wrote the software for angle analysis and wrote the data processing sections.

## Pre-publication history

The pre-publication history for this paper can be accessed here:


